# Hypervirulent *Klebsiella pneumoniae* Strains Modulate Human Dendritic Cell Functions and Affect T_H_1/T_H_17 Response

**DOI:** 10.3390/microorganisms10020384

**Published:** 2022-02-07

**Authors:** Sabrina Nicolò, Giorgio Mattiuz, Alberto Antonelli, Fabio Arena, Vincenzo Di Pilato, Tommaso Giani, Ilaria Baccani, Ann Maria Clemente, Giuseppe Castronovo, Michele Tanturli, Federico Cozzolino, Gian Maria Rossolini, Maria Gabriella Torcia

**Affiliations:** 1 Department of Experimental and Clinical Medicine, University of Florence, 50134 Florence, Italy; sabrina.nicolo@unifi.it (S.N.); giorgio.mattiuz@unifi.it (G.M.); alberto.antonelli@unifi.it (A.A.); tommaso.giani@unifi.it (T.G.); ilaria.baccani@unifi.it (I.B.); annmariaclemente@virgilio.it (A.M.C.); gianmaria.rossolini@unifi.it (G.M.R.); 2 Clinical Microbiology and Virology Unit, Careggi University Hospital, 50134 Florence, Italy; vincenzo.dipilato@unige.it; 3 Department of Clinical and Experimental Medicine, University of Foggia, 71122 Foggia, Italy; fabio.arena@unifg.it; 4 IRCCS Fondazione Don Carlo Gnocchi ONLUS, 50143 Florence, Italy; 5 Department of Surgical Sciences and Integrated Diagnostics, University of Genoa, 16132 Genoa, Italy; 6 Department of Experimental and Clinical Biomedical Sciences “Mario Serio”, University of Florence, 50139 Florence, Italy; giuseppe.castronovo@unifi.it (G.C.); michele.tanturli@unifi.it (M.T.); federico.cozzolino@unifi.it (F.C.)

**Keywords:** hypervirulent, hypermucoviscous *K. pneumoniae*, dendritic cells (DCs), T_H_ differentiation, inflammatory cytokines, immune response

## Abstract

Hypervirulent *Klebsiella pneumoniae* (Hv-Kp) strains have emerged as pathogens causing life-threatening, invasive disease even in immunocompetent hosts. Systemic dissemination usually occurs following perturbations of the gut microbiota and is facilitated by Hv-Kp resistance to phagocytosis and complement activity. Hv-Kp are usually associated with K1 or K2 capsular types, produce several iron uptake systems (e.g., aerobactin and salmochelin) and are often but not invariably, capsular material hyper-producers (hypermucoviscous phenotype: HMV). Whether Hv-Kp escape the immune response at mucosal site is unknown. In this work, we studied the effects of Hv-Kp on human dendritic cells (DCs), central players of the IL-23/IL-17 and IL-12/IFN-γ axis at mucosal sites, essential for pathogen clearance. Four Hv-Kp and HMV strains were selected and their activity on DC maturation and cytokine production was compared to that of non-virulent Kp strains with classic or HMV phenotypes. While the maturation process was equally induced by all Kp strains, significant differences between virulent and non-virulent strains were found in the expression of genes for cytokines involved in T-cell activation and differentiation. The non-virulent KP04C62 and the classic Kp, KPC157 induced high expression of T_H_1 (IL-12p70 and TNFα) and T_H_17 cytokines (IL-23, IL-1β and IL-6), while Hv-Kp poorly activated these cytokine genes. Moreover, conditioned media from DCs cultured with non-virulent Kp, either classical or hypercapsulated, induced the activation of IL-17 and IFN-γ genes in preactivated CD4^+^-cells suggesting their T_H_17/T_H_1 differentiation. Conditioned media from Hv-Kp poorly activated IL-17 and IFN-γ genes. In summary, our data indicate that Hv-Kp interfere with DC functions and T-cell differentiation and suggest that the escape from the IL-23/IL-17 and IL-12/IFN-γ axes may contribute to pathogen dissemination in immunocompetent hosts.

## 1. Introduction

*Klebsiella pneumoniae* (Kp), known as an opportunistic pathogen with a high propensity to acquire resistance genes, is a frequent cause of severe nosocomial infections in immunocompromised patients, mainly pneumonia, urinary tract infections and bacteremia [1,2]. In hospitalized patients, *K. pneumoniae* colonizes the intestinal microbiota, and its establishment in this environment is considered a fundamental step for the development of subsequent infections at distant body sites [3,4,5]. Since the mid-1980s, peculiar Kp lineages, denominated hypervirulent Kp (Hv-Kp), have emerged worldwide and are responsible for pyogenic infections with metastatic dissemination (e.g., liver abscesses, osteomyelitis, and endophthalmitis) [6], even in immunocompetent hosts [7,8].

Although we are still far from a universally accepted definition of Hv-Kp, some genetic features are considered hallmarks of virulent strains (Table 1) and typically consist of accessory virulence factors coding for (i) regulators of the mucoid phenotype, including *rmpA* and/or *rmpA2,* and the recently described *rmpD* gene necessary for hypermucoviscosity and virulence [9]; (ii) siderophore systems, including aerobactin (*iucABCD*), allantoin (*allABCDRS*), colibactin (*clbA-QS*), yersiniabactin (*ybtAEPQSTUX-irp1/2-fyuA*) and salmochelin (*iroBCDEN*) biosynthesis loci [10]. Additional virulence factors may consist of genes coding for proteins involved in iron metabolism (*cobW*) and transport (*fecI*-*fecA*), the hemin and lysine transport system (*shiF*), metabolic transporter (*peg-344*), and transcriptional regulations of virulence gene expression (*luxR*) [11].

Interestingly, all *K. pneumoniae* also harbor an array of core chromosomally located pathogenicity factors, including the siderophore enterobactin (*entABCDEF*-*fepABCDG*), as well as genetic loci encoding type 1 (*fim*) and type 3 (*mrk*) fimbriae and the variable capsular (cps) polysaccharide (K antigen) [10,12,13]. Different from “classical” Kp strains, Hv-Kp, show high virulence potential in animal experimental models (particularly those with K1 and K2 cps types and accessory siderophore systems) and usually belong to specific clonal lineages, such as clonal groups (CGs) 23, CG86 and CG65, retaining susceptibility against most of available antibiotics [13,14].

Hv-Kp, usually colonize the gut microbiota [15] and gain access to other sterile sites in the host following the impairment of microbiota resistance to colonization and dysbiosis-induced leakage of the epithelial barrier [16].

Some hypermucoviscous phenotype (HMV) Kp strains that are negative for *rmpA*/*rmpA2* and other genotypic markers of virulence were also reported to be able to cause disseminated infections in immunocompromised patients [13,14,16,17]. These strains, however, do not show pathogenic potential in experimental models of infections suggesting that, despite the hyperproduction of capsular polysaccharides, they are efficiently cleared by healthy immune-competent human hosts at mucosal sites.

Recent evidence shows that the HMV phenotype is not essential for the persistence of Hv strains in the gut [18], suggesting that mechanisms of immune escape that are not dependent on phagocytosis resistance, cooperate for pathogen survival at the mucosal level.

Dendritic cells (DCs) at mucosal sites capture and process antigens of microbiota components and, when necessary, orchestrate the activation of T cells and innate lymphoid cells (ILCs). An efficient adaptive response against Kp requires the integrity of the IL-23a/IL-17 and IL-12a/IFN-γ axes [19,20] and thus the full functionality of DCs.

In this paper, we selected four HMV strains originally isolated from disseminated infections and studied their effects on DC functions and T-cell differentiation. The hypervirulent HMV CIP 52.145 and a “classical” multi-resistant Kp strain were used as reference strains.

## 2. Materials and Methods

### 2.1. Bacterial Strains

CIP 52.145 is an Hv/HMV-Kp, multi-susceptible, well-characterized strain, isolated from a human specimen in Indonesia. It has a K2 cps type and belongs to ST66 [21,22].

RM1628 is an Hv/HMV-Kp, multi-susceptible, clinical isolate obtained in Italy from the blood culture of a patient with a liver abscess [23]. It has a K1 cps type and belongs to an ST related to the well-characterized Hv-Kp ST23.

HMV-1 and HMV-2 are two Hv/HMV-Kp, multi-susceptible, clinical isolates obtained from bloodstream infections in Italy. They belong to ST86 and ST65, respectively, and have a K2 cps type [24].

KP04C62 is an HMV-Kp, carbapenem-resistant, clinical isolate, obtained in Italy from the blood culture of a severely immunocompromised patient with a liver abscess. It belongs to ST512 and is associated with the KL107 cps type [17].

KPC157 is a “classical”, non-HMV, carbapenem-resistant, Kp strain obtained from a rectal swab of a colonized patient, in Italy. It belongs to ST512 and is associated with the KL107 cps type [24] (Table 1).

The genomic sequences of studied strains were previously deposited and are accessible at the National Center for Biotechnology Information (https://www.ncbi.nlm.nih.gov/, accessed on 14 December 2021).

Screening for known *K. pneumoniae* virulence genes, including the recently described *rmpD* was performed using the Basic Local Alignment Search Tool (BLAST) (https://blast.ncbi.nlm.nih.gov/Blast.cgi, accessed on 14 December 2021) and the Virulence Factor Database (VFDB, [9,11,22,25]).

Data on virulence in the animal model were reported accordingly to previously published results [17,23,24]. The string test was performed as previously described [17]. A summary of the genetic, phenotypic, and virulence features of the studied strains is shown in Table 1.

**Table 1:** Characteristics of Kp strains included in this work.

	Source	Mucoid Phenotype (String Test)	Capsular Type (O Locus)	Sequence Type (ST)	Antibiotic Resistance	Virulence in Animal Model	Aerobactin (*iucABCD*)	Allantoin (*allABCDRS*)	Colibactin (*clbA-QS*)	Enterobactin (*entABCDEF-fepABCDG*)	Yiersiniabactin (*ybtAEPQSTUX*-irp1/2-fyuA)	Salmochelin (*iroBCDEN*)	Reg. Mucoid Phenotype (*rmpADC-rmpA2*)	*manBC*	*shiF*	*fecI-fecA*	*peg-344*	*luxR*	pK2044-like Plasmid	Genome Accession Number	Ref.
CIP 52.145	Human	Positive	K2 (O2v2)	66	Multi-susceptible	++			#				¤						*	GCA_000968155.1	[21,22]
RM1628	Human, BSI	Positive	K1 (O1v2)	1861	Multi-susceptible	++														JAALJC000000000.1	[23]
HMV-1	Human, BSI	Positive	K2 (O1v1)	86	Multi-susceptible	++							¤							JAALCW000000000	[24]
HMV-2	Human, BSI	Positive	K2 (O1v2)	65	Multi-susceptible	++														JAALCV000000000	[24]
KP04C62	Human, BSI	Positive	KL107 (O2v2)	512	Carbapenem-resistant	+/−						§								MIFX00000000.1	[17]
KPC157	Human, RS	Negative	KL107 (O2v2)	512	Carbapenem-resistant	+/−						§								JAALCU000000000.1	[24]

Legend: ++: high virulence comparable to that NTUH-K2044 reference strain; +/−: low virulence comparable to that of other classical *K. pneumoniae* strains; the presence of virulence genes is highlighted in green, while their absence in red; BSI, bloodstream infection; RS, rectal swab; # absence of *clbK*; § presence of *iroE* only; ¤ absence of *rmpA2*; * coverage 33%, identity 98.95%.

### 2.2. Ethical Approval

The use of buffy coats from donated blood, not usable for therapeutic purposes, was approved by the Ethics Committee of the Azienda Ospedaliera Universitaria Careggi (AOUC, Firenze, Italy) in agreement with the D.M. of the Italian Ministry of Health (15A09709) G.U., n. 300 12.28. 2015).

### 2.3. Cell Isolation Procedures

Peripheral blood mononuclear cells (PBMCs) were isolated by buffy coats through gradient centrifugation using Ficoll-Paque (GE Healthcare Italia, cat #45-001-750), according to the manufacturer’s recommendations. CD14^+^-cells were isolated using anti-CD14 conjugated microbeads (Miltenyi Biotec, cat #130-050-201) [26]. Monocyte-derived dendritic cells (DCs) were obtained by stimulating CD14^+^ cells with recombinant IL-4 (50 ng/mL) and recombinant GM-CSF (100 ng/mL) [27].

CD4^+^ T-cells were isolated from a non-adherent fraction of PBMCs by using a CD4^+^ T-cells separation kit (Miltenyi, cat #130-096-533) according to the manufacturer’s recommendations. The purity of the populations was checked using cytofluorimetric analysis with specific antibodies and was always >90%.

### 2.4. T_H_1 and T_H_17 Differentiation

Purified CD4^+^T-cells from different donors were pre-activated by anti-CD3/CD28 antibodies coupled to beads at a 1:1 bead/cell ratio (Gibco, cat #11131D).

Preactivated CD4^+^ T-cells (10^6^/mL) were then incubated with 1 mL of conditioned medium obtained from cultures of DCs with Kp strains following the methods of Santini et al. [28]. Cytokine gene expression was evaluated by real -time (RT) PCR.

### 2.5. Cell Culture Conditions

In all experiments DCs and T-cells were cultured with live bacteria (50 MOI/cell) in an RPMI medium supplemented with 10% fetal bovine serum (Celbio, cat # 26140) and 5% L-glutamine (complete medium, CM), at 37 °C in a humidified chamber with 5% CO_2_.

### 2.6. Western Blot Analysis

A quantity of 10^6^ DCs was cultured in CM for 30 min in the presence or absence of live bacterial cells (50 MOI/cell) or 200 ng/mL of pure LPS (Sigma-Aldrich, Saint Louis, MO, USA, cat. #L4391). Cells were lysed with RIPA buffer in the presence of phosphatase/protease inhibitor cocktail (Sigma-Aldrich, Saint Louis, MO, USA) and centrifuged at 12,000× *g* for 15 min, and the protein concentration was determined by BCA assay (Quantum Protein Assay Kit, EuroClone Pero, Italy). Subsequently, 40 μg of proteins/lane were loaded onto Stain Free gel (Bio-Rad Hercules, CA, USA) SDS-PAGE and blotted onto nitrocellulose filters (Bio-Rad, Hercules, CA, USA).

Membranes were stained with rabbit anti-phospho-NF-κB (p65) and anti phospho-p38 MAPK (Cell Signaling Technology, Danvers, MA, USA) antibodies at a 1:1.000 final dilution. Anti-rabbit IgG (H+L) DyLight800 were used as secondary antibodies at a1:10.000 final dilution. The reactions were visualized with the ECL detection system as recommended by the manufacturer (Bio-Rad Hercules, CA, USA). The intensity of the total proteins on the membrane was acquired by stain-free technology (Bio-Rad Hercules, CA, USA) using the ChemiDoc Touch System (Bio-Rad Hercules, CA, USA). The densitometric analysis was expressed as the ratio between the protein of interest and the total proteins by Image Lab software (Bio-Rad Hercules, CA, USA). [29]

### 2.7. Cytofluorimetric Analysis

Live bacterial cells were incubated with 5 × 10^5^ DCs as reported above, for 16 h. At the end of the incubation cells were washed and stained with a mixture of anti-CD83-FITC, anti-CD86-APC and anti-HLA-DR-PE antibodies (BD Biosciences-Pharmingen, cat. #560929; #555660, and #555812) for 30 min. Cells were analyzed using the ACCURI instrument (BD Biosciences, Franklin Lakes, NJ, USA) using Cflow Plus software (BD Biosciences, Franklin Lakes, NJ, USA). Ten thousand events for each sample were acquired.

### 2.8. Real-Time PCR

T-cells and DCs cells (10^6^) were cultured in presence of Kp strains for 3 h and 3 days, respectively. RNA extraction was performed using TRIzol™ (Invitrogen, cat. #15596-018). Extracted RNAs were quantified using Nanodrop (Thermo Fisher, Waltham, MA, USA) and stored at −80 °C.

A total of 2 μg of RNA from each sample was reverse-transcribed using EuroScript M-MLV Reverse Transcriptase (RNase H-) (EuroClone, cat. #EMR437050). RT-PCR was performed by using the QuantiNova SYBR Green PCR kit (Qiagen, cat. #208056) and the 7900HT Fast Real-Time PCR System (Applied Biosystems™). A total of 50 ng of cDNA from each sample was amplified. The β-actin gene was used as housekeeping gene. The primers used in this work are reported in Table 2.

### 2.9. Cytokine Production

We measured the IL-12p70 and IL-1β concentrations in the supernatant derived from cultures of 1 × 10^6^ DCs incubated with Kp strains for 16 h. These data were produced using the Milliplex^®^ Map Human Cytokine kit and Luminex apparatus following the manufacturer’s instructions (Luminex 200 MAGPIX).

### 2.10. Statistical Analysis

RT-PCR data were analyzed using the Kruskal-Wallis test and analysis of variance (ANOVA) from three different experiments. Bonferroni p-value adjustment method for multiple comparisons was used. A probability value of *p* < 0.05 was considered significant. Statistical analysis was performed using R software version 3.6.1 [30]. Western blot statistical analysis was performed by paired t-test.

## 3. Results

### 3.1. Expression of Maturation Markers

**Figure 1:**
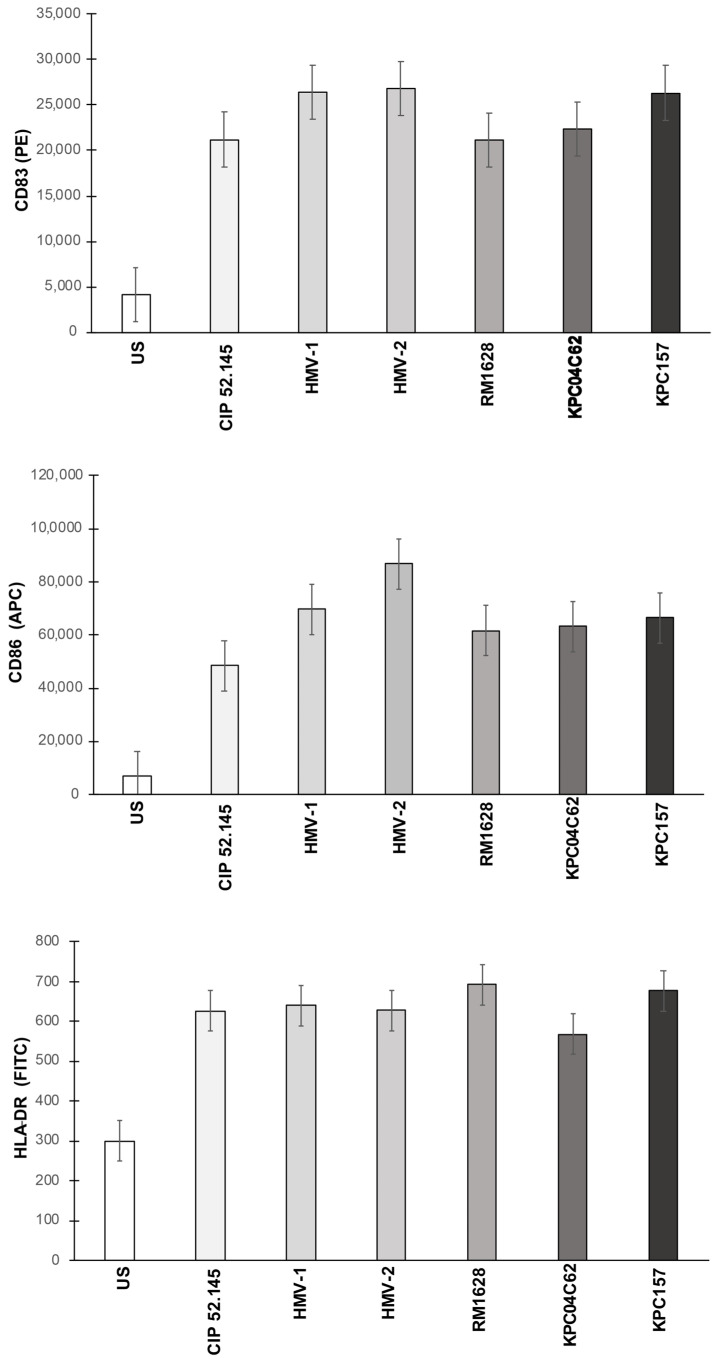
Maturation of DCs induced by *K. pneumoniae* strains. DCs were cultured with medium alone (unstimulated, US) or with live bacterial cells. Data were collected with a cytofluorimeter and are expressed as median fluorescence intensity (± IQR ×1.5) of three different experiments. The maturation level of each sample was compared (with the exception of US) and no significant statistical differences were observed.

The cytofluorimetric analysis of CD83, CD86 and HLA-DR expression on DCs showed that all Kp strains induced a robust expression of CD83 and CD86 determinants on the membranes of DCs (Figure 1).

The expression of HLA-DR was also induced by all Kp strains compared with their uninfected counterparts. Statistical analysis did not reveal significant differences in the expression of these markers on DCs (valuated as median fluorescence expression) among DCs cultured with different Kp strains. Histograms of one experiment out of the three performed are shown in Supplementary Figure S1.

### 3.2. Cytokine Gene Expression by DCs Infected with Kp Strains

**Figure 2:**
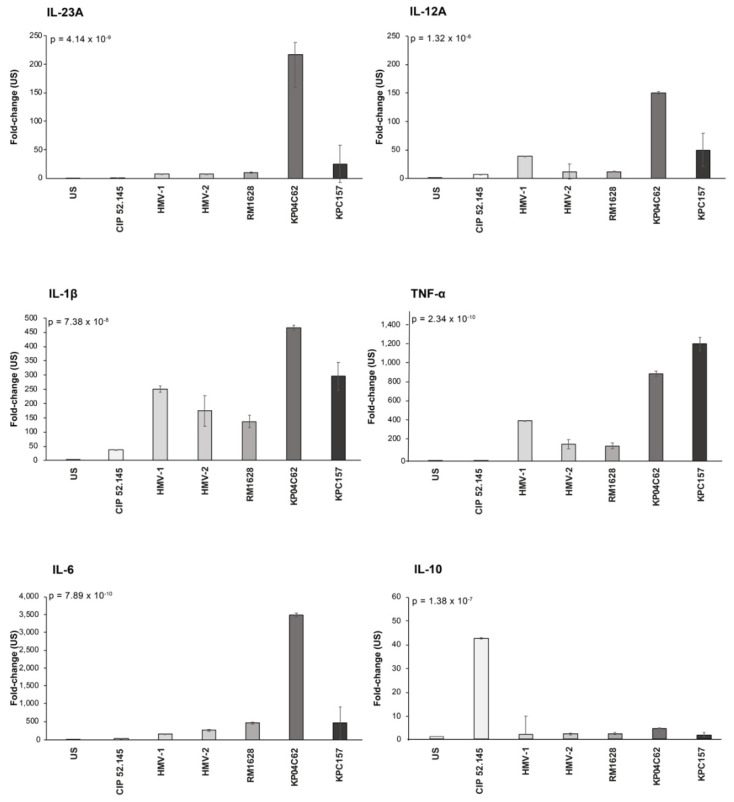
Cytokine expression by DCs cultured in presence of live *K. pneumoniae* strains; the global *p*-value obtained by ANOVA is reported.

The expression of cytokines involved in T_H_17 (IL-1β, IL-23a, and IL-6) and T_H_1 differentiation (IL-12a, and TNF-𝛼) and the anti-inflammatory response (IL-10) was investigated. Figure 2 and  Figure S2 show the cytokine gene expression induced by Kp strains in DCs. Two main clusters were detectable on the basis of cytokine gene activation. The first included RM1628, HMV-1, HMV-2, and the reference Hv-Kp CIP 52.145. All these strains induced very low or no expression of all cytokine genes evaluated, with selected cytokine genes activated less than control, unstimulated cultures. The second cluster included KP04C62 and the classical KPC157. These two Kp strains activated all cytokine genes included in the study.

The histograms presented in Figure 2 show a more detailed graphical representation of the effects of each Kp strain on cytokine gene activation. It should be noted that the HMV non-virulent KP04C62 strain had similar behavior to the classical Kp strain, and both strains activated DCs to express cytokine genes involved in the T_H_17 (IL-23, IL-6, and IL-1β) and T_H_1 (IL-12 and TNF-α) responses (results of post hoc tests are reported in Supplementary Table S1).

In contrast, the clinical isolates HMV1 HMV2 RM1628 and the reference virulent CIP 52.145 only slightly activated these genes. The statistical analysis revealed significant differences between Kp strains, particularly when comparing the results of virulent strains with those obtained from classical KPC157 or hypercapsulated KP04C62 strains.

Moreover, Figure 2 shows that the reference CIP 52.145 was the most potent inducer of the anti-inflammatory IL-10 gene expression. Apart from CIP 52.145, the expression of the IL-10 gene was not significantly induced by Kp strains, virulent or not, during 6 h of stimulation, suggesting that the activation of this immunomodulatory pathway may not be included in the escape strategy of virulent Kp.

We measured the concentrations of IL-1β and IL-12p70 in the supernatants from DCs cultured with *K. pneumoniae* strains. Supplementary Table S2 shows that despite the prolonged time of stimulation, IL-12 concentrations were in accordance with data from gene expression, while IL-1β was produced in high amounts by one of the Hv-Kp strains.

### 3.3. T_H_1 and T_H_17 Differentiation Induced by Kp-Conditioned Media of DCs Cultures

**Figure 3:**
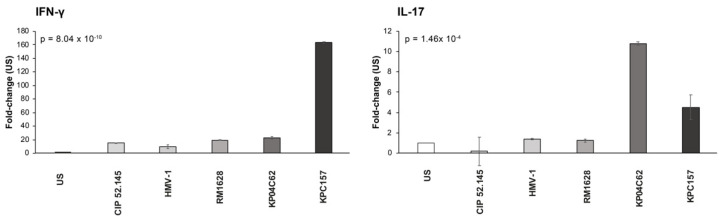
IFN-γ and IL-17 gene expression by pre-activated CD4^+^ T-cells. The bar-graph shows data (mean ± SE) of three different experiments. Data are expressed as fold-change with respect to unstimulated (US) cultures; the global *p*-value obtained by ANOVA is reported.

To add further evidence of the inhibitory effects of HMV-Kp strains on DC functions, we assessed conditioned media from Kp-DC cultures for the ability to induce the differentiation of pre-activated CD4^+^ T-lymphocytes. Figure 3 and Supplementary Table S3 show that the conditioned media from DCs cultured with HMV-Kp strains were ineffective at inducing IL-17 gene expression of pre-activated CD4^+^ cells. By contrast, the conditioned media from DCs cultured in presence of non-virulent Kp strains, either classical (KPC157), or hypercapsulated (KP04C62), strongly induced IL-17 gene expression by pre-activated CD4^+^ T-cells. The medium of DCs cultured with classical Kp was also able to induce a significant amount of IFN-γ gene expression. The data suggest that virulent strains of Kp may affect DC functionality and compromise the differentiation of T_H_17 and T_H_1 effector T cells.

### 3.4. Molecular Mechanisms of Inhibition of Cytokine Gene Expression

The nuclear translocation of AP-1 and NF-kB (p65) following phosphorylation mediated by p38-MAPK and TRAF6 respectively [31] represents a crucial step for the transcription of cytokine genes.

**Figure 4:**
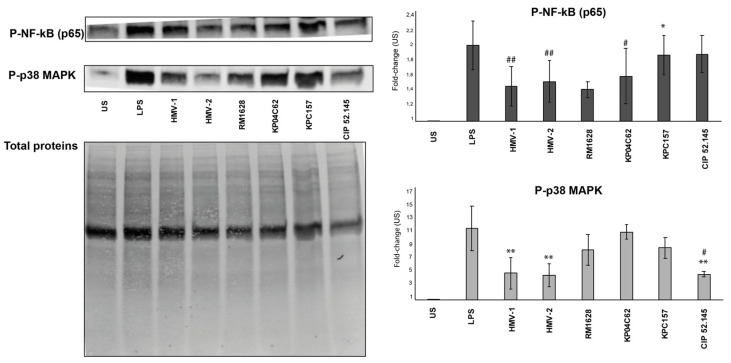
NF-kB (p65) activation and p38-MAPK phosphorylation by DCs. Dendritic cells were cultured in the absence (US) or presence of *K. pneumoniae* strains or LPS as a positive control. Cells were analyzed by Western blot analysis. Data from one representative experiment out of three performed are shown. Data are expressed as the fold increase of each experimental point over unstimulated control. A *t*-test was performed comparing KP04C62 (*) and KPC157 (#) (non-Hv-HMV strains) with the Hv-HMV Kp strains. We considered statistical significance as a *p*-value < 0.05 (*, #); *p*-value < 0.01, (**, ##).

To investigate whether Hv-HMV Kp strains affect these pathways, DCs were cultured with live bacterial cells for 30 min and the activation of NF-kB and p38MAPK was measured using Western blot analysis. Figure 4 shows that the activation of p38-MAPK was significantly reduced in cells challenged with HMV-Kp compared with those challenged with KP04C62 and KPC157, suggesting that the HMV phenotype may interfere with the activation of pro-inflammatory pathways in DCs.

## 4. Discussion

*K. pneumoniae* is considered a stealth pathogen because it fails to stimulate the innate immune response [24]. These bacteria, in fact, have evolved numerous mechanisms to avoid recognition from host pattern recognition receptors (PRRs). Much evidence, however, indicates that *Klebsiella* also actively subverts host defenses. For example they are able to manipulate phagosome maturation or modify lipid A decoration [19,20].

Hv-Kp shares many of these mechanisms with classical Kp. The HMV phenotype allows pathogens to escape neutrophil phagocytosis and complement-mediated activity and is crucial for their systemic dissemination.

Hv-Kp usually colonize the gastrointestinal (GI) tract [15], and, under certain circumstances (as for example, antibiotic-induced dysbiosis), they may spread to other organs and apparatus [16,32,33].

The persistence of Hv-Kp in the gut, however, is not strictly associated with capsule hyperproduction and the HMV phenotype [18], and it is reasonable that Hv-Kp escapes other mechanisms of innate and adaptive immunity.

It is well known that DCs are important players of mucosal immunity, as they sense the external environment, capture living or dead bacterial cells or single antigens released after cell death, and present antigens to T cells, supporting their activation [34]. Bacterial ligands interacting with PRRs localized in the cytoplasmic membrane, endosomes or cytosol of DCs promptly activate the expression of membrane determinants involved in immunological synapsis as well as the production of cytokines responsible for Th differentiation [35]. Numerous TLRs and both TRIF- and MyD88-dependent signaling contribute to host defense against *K. pneumoniae* infection [36].

In addition, capsular polysaccharides from either classical or HMV Kp activate the NLRP3 inflammasome pathway, leading to mature IL-1 beta and IL-18 production [37,38]. These cytokines activate and potentiate IL-12 and IL-23 functions, driving the differentiation of T_H_ precursors T_H_1 and T_H_17 activated T-cells [39].

DCs also activate innate group 3 lymphoid cells (ILC3) during infection sustained by members of the *Enterobacteriaceae* family [40], producing a variety of cytokines, including IL-22 and IL-17A, and providing crucial protection against enteric pathogen infections.

The main results of our study show that Hv-Kp strains interfere with DC expression of IL-12 and IL-23 and, as a result, DCs lose their ability to induce the differentiation of T-cells to T_H_1 or T_H_17 effectors. This behavior can potentially compromise the adaptive immune response and the clearance of these pathogens. All Kp strains, virulent or not, were able to induce the expression of surface MHC class II and other accessory molecules (CD83 and CD86), suggesting their ability to activate toll like receptor (TLR) 4 and other membrane or cytosolic PRRs. Moreover, preliminary data suggest that DCs cultured with Hv-Kp do not undergo apoptosis, suggesting that TLR pathways are activated and maintain DC viability. As such, the abundance of capsular polysaccharides is not the only cause of reduced cytokine gene activation. Consistent with this hypothesis, the non virulent HMV included in this study, KP04C62, was found to not interfere with the activation of cytokine genes.

Interestingly, it has been recently reported that Hv-Kp strains are able to resist thekilling activity of human macrophages and survive within the cells [23]. Rather than low interaction with TLRs, the molecular mechanisms of the inhibitory activity exerted by Kp more likely reside in interference with the NF-kB and MAPK signaling pathways. Our data indeed show that the activation of p38MAPK and NFkB pathways is significantly lower in DCs cultured with the Hv-Kp strains compared with that in DCs cultured with not virulent Kp, suggesting that Hv-Kp produce factors that interfere with these signaling pathways. Such interference was reported in different experimental systems [41,42] and was attributed to bacterial components, such as the LPS O-polysaccharide and PulA type 2 secretion system [43].

Genetic analysis of the strains included in the study revealed a heterogeneous content of core and accessory genes linked to virulence, which might contribute to the different behavior shown in activation of p38MAPK and NF-kB pathways by Hv-Kp strains.

Further studies are needed to clarify which factors are responsible for DC inhibition.

In accordance with the low amount of IL-12/IL-23, we observed that conditioned media from DCs cultured with Hv-Kp do not induce the T_H_1/T_H_17 differentiation of preactivated CD4+ cells, suggesting that the activation of the above mechanisms may not take place in vivo.

As reported above, an efficient adaptive response against Kp requires the integrity of the IL-23a/IL-17 and IL-12a/IFN-γ axes [19,41,42] and the full functionality of DCs. T_H_1 and T_H_17 cells support the clearance of Kp by inducing neutrophil recruitment and activation, the production of neutrophil extracellular traps (NETs) and the IFN-γ mediated potentiation of macrophage activity [41]. Along with the crucial role in the activation of the IL-23a/IL-17 and IL-12a/IFN-γ axes, DCs also stimulate innate lymphoid cells at mucosal sites to produce IL-22, a cytokine promoting gut homeostasis through its functional effect on the epithelial barrier [43]. The functional inactivation of DCs by Hv-Kp is suggested as a major cause of systemic dissemination of these pathogens even in healthy, non immune-compromised patients.

However, further experiments are needed to define the factor(s) involved in the inhibition of p38/NF-kB activated pathways. Our data underscore the necessity to formulate vaccines against proteinaceous (non-capsular) Kp antigens, which, by expanding the population of memory-resident T_H_17 lymphocytes in the mucosal districts, also guarantee an optimal adaptive response to infections by antibiotic-resistant strains.

## 5. Conclusions

The results of this study indicate that the interference of Hv-Kp with the functions of DCs affects the differentiation of T-cells to T_H_17 and T_H_1 effectors. These findings provide evidence for the ability of Hv-Kp to escape the host adaptive response at mucosal sites and potentially disseminate in immunocompetent hosts.

## Figures and Tables

**Table 2:** Primers used in this work.

Gene	Forward 5′-3′	Reverse 5′-3′
B-actin	GAAACTACCTTCAACTCCATCATG	AGGAGGAGCAATGATCTTGATC
IL-23a	CTCAGGGACAACAGTCAGTTC	ACAGGGCTATCAGGGAGCA
IL-12a	CCTTGCACTTCTGAAGAGATTGA	ACAGGGCCATCATAAAAGAGGT
IL-1β	AGCTACGAATCTCCGACCAC	CGTTATCCCATGTGTCGAAGAA
TNF-α	CCTCTCTCTAATCAGCCCTCTG	GAGGACCTGGGAGTAGATGAG
IL-6	ACTCACCTCTTCAGAACGAATTG	CCATCTTTGGAAGGTTCAGGTTG
IL-10	TCAAGGCGCATGTGAACTCC	GATGTCAAACTCACTCATGGCT
IL-17	AGATTACTACAACCGATCCACCT	GGGGACAGAGTTCATGTGGTA
IFN-γ	TCGGTAACTGACTTGAATGTCCA	TCGCTTCCCTGTTTTAGCTGC

## Data Availability

Data is contained within the article or supplementary material.

## Supplementary Materials

The following are available online at https://www.mdpi.com/article/10.3390/microorganisms10020384/s1, Table S1: ANOVA test with Holm *p*-value adjustment for each *Klebsiella pneumoniae* stimulus. Table S2: Evaluation of Cytokine’s production by DCs under bacteria stimulation. The concentration reported refers to the amounts from three different cultures pooled together. US indicates the control, unstimulated cultures. Figure S1: DCs maturation under bacteria stimuli was evaluated by FACS analysis. DCs were stained with a mixture of anti-HLRA-DR-PE (upper lane), anti-CD86-APC (middle lane) and anti-CD83-FITC (lower lane) antibodies. Red gate indicates P1 population (live cells), blue gate to P2 population (we exclude doublets). The gate for the analysis was based on the US (vertical black line). Figure S2: Heat-map of the expression of cytokine in stimulated DCs. Genes are reported on x-axis and Kp stimuli on the y-axis.
