# Supplementary material for: Hypervirulent Klebsiella pneumoniae Strains Modulate Human Dendritic Cell Functions and Affect TH1/TH17 Response
Source: Microorganisms. 2022 Feb 7;10(2):384. doi: 10.3390/microorganisms10020384 (PMC8877041; doi:10.3390/microorganisms10020384)
Supplement: Supplementary file 1 [file microorganisms-10-00384-s001.zip › microorganisms-1535380 -supplementary.pdf]

**IL-23A**	US	LPS	CIP 52.145	HMV-1	HMV-2	RM1628	KP0C62
LPS	1	-	-	-	-	-	-
CIP 52.145	1	-	-	-	-	-	-
HMV-1	1	1	-	-	-	-	-
HMV-2	1	1	1	-	-	-	-
RM1628	1	1	1	1	-	-	-
KP04C62	0.000000013	1.3E-08	0.000000013	1.9E-08	1.9E-08	2.1E-08	-
KPC157	1	1	1	1	1	1	0.000000061

**IL-1β**	US	LPS	CIP 52.145	HMV-1	HMV-2	RM1628	KP0C62
LPS	1	-	-	-	-	-	-
CIP 52.145	1	1	-	-	-	-	-
HMV-1	0.00053	0.00079	0.00232	-	-	-	-
HMV-2	0.01566	0.02233	0.06694	0.55758	-	-	-
RM1628	0.06694	0.0978	0.22882	0.14017	1	-	-
KP04C62	0.00000025	3.3E-07	0.00000072	0.00232	0.00009921	0.00002442	-
KPC157	0.00009405	0.00013	0.00037	1	0.11029	0.00261	0.01645

**IL-6**	US	LPS	CIP 52.145	HMV-1	HMV-2	RM1628	KP0C62
LPS	1	-	-	-	-	-	-
CIP 52.145	1	1	-	-	-	-	-
HMV-1	1	1	1	-	-	-	-
HMV-2	1	1	1	1	-	-	-
RM1628	1	1	1	1	1	-	-
KP04C62	1.9E-09	1.9E-09	2.1E-09	3.5E-09	5.3E-09	1.31E-09	-
KPC157	1	1	1	1	1	1	1.31E-09

**IFN-!**	US	CIP 52.145	HMV-1	RM1628	KP0C62
CIP 52,145	0.9	-	-	-	-
HMV-2	1	1	-	-	-
RM1628	0.38	1	1	-	-
KP04C62	0.19	1	0.9	1	-
KPC157	1.7E-09	4E-09	2.9E-09	5.3E-09	6.4E-09

**IL-12A**	US	LPS	CIP 52.145	HMV-1	HMV-2	RM1628	KP0C62
LPS	1	-	-	-	-	-	-
CIP 52,145	1	1	-	-	-	-	-
HMV-1	0.54774	0.54774	0.9216	-	-	-	-
HMV-2	1	1	1	1	-	-	-
RM1628	1	1	1	1	1	-	-
KP04C62	2.7E-06	2.7E-06	5E-06	0.00011	7.1E-06	7.1E-06	
KPC157	0.17302	0.17302	0.3571	1	0.56097	0.56097	0.00033

**TNF-!**	US	LPS	CIP 52.145	HMV-1	HMV-2	RM1628	KP0C62
LPS	1	-	-	-	-	-	-
CIP 52,145	1	1	-	-	-	-	-
HMV-1	0.00188	0.00188	0.0019	-	-	-	-
HMV-2	0.50699	0.50699	0.507	0.10526	-	-	-
RM1628	0.52163	0.52163	0.5216	0.07485	1	-	-
KP04C62	2E-07	2E-07	2E-07	0.00046	3.12E-06	2.42E-06	-
KPC157	3.4E-09	3.4E-09	3E-09	1.15E-06	2.99E-08	2.45E-08	0.02609

**IL-10**	US	LPS	CIP 52.145	HMV-1	HMV-2	RM1628	KP0C62
LPS	1	-	-	-	-	-	-
CIP 52.145	4E-06	6.9E-07	-	-	-	-	-
HMV-1	1	1	5E-07	-	-	-	-
HMV-2	1	1	5E-07	1		-	-
RM1628	1	1	5E-07	1	1	-	-
KP04C62	1	1	1E-07	1	1	1	-
KPC157	1	1	5E-07	1	1	1	1

**IL-17**	US	CIP 52.145	HMV-1	RM1628	KP0C62
CIP 52.145	1	-	-	-	-
HMV-2	1	1	-	-	-
RM1628	1	1	1	-	-
KP04C62	0.00124	0.00021	0.0006	0.00053	-
KPC157	1	0.13777	0.4779	0.44699	0.01492

Supplementary Table S1. In tables are reported ANOVA test with Holm p value adjustment for each *Klebsiella pneumoniae* stimuli.

Cytokine production (pg/mL)		
	IL-12p70	IL-1β
US	14	6
CIP 52.145	73	355
HMV-1	185,8	435
HMV-2	217,75	622
RM1628	238,5	266,5
KP04C62	1434	185,5
KPC157	746	672

Supplementary Table S2. Cytokine's production of DCs. Equal amounts from three different cultures were pooled. US: unstimulated cultures.

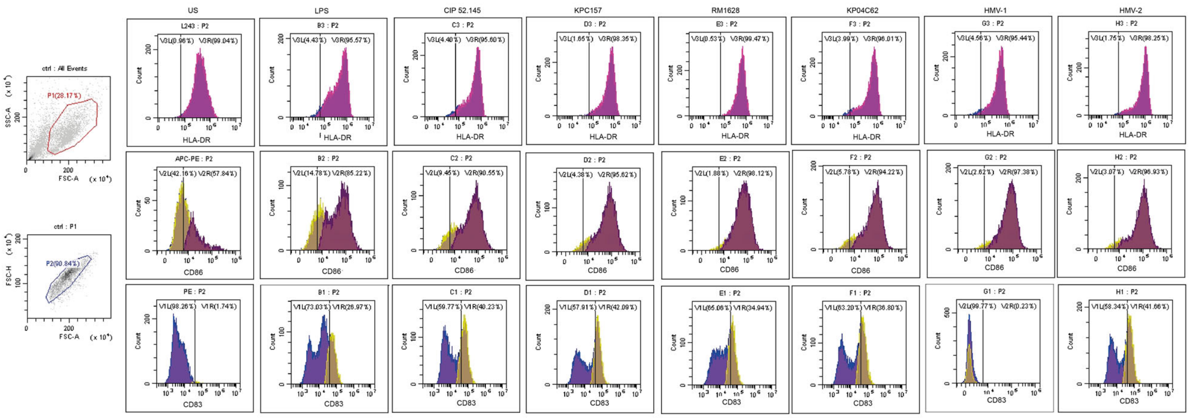

Supplementary Figure S1. DCs were stained with a mixture of anti-CD83-FITC, anti-CD86-APC and anti-HLRA-DR-PE antibodies. Ten thousand events for each sample were acquired. US: unstimulated.

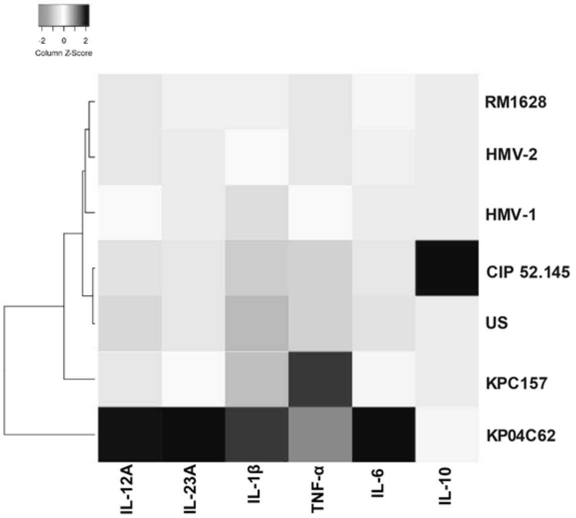

Supplementary Figure S2. Cytokine expression by DCs cultured in presence of live *K. pneumoniae* strains by heat-map analysis.
